# Meaningful activities during COVID-19 lockdown and association with mental health in Belgian adults

**DOI:** 10.1186/s12889-021-10673-4

**Published:** 2021-03-30

**Authors:** Ellen Cruyt, Patricia De Vriendt, Miet De Letter, Peter Vlerick, Patrick Calders, Robby De Pauw, Kristine Oostra, Maria Rodriguez-Bailón, Arnaud Szmalec, Jose Antonio Merchán-Baeza, Ana Judit Fernández-Solano, Laura Vidaña-Moya, Dominique Van de Velde

**Affiliations:** 1 Faculty of Medicine and Health Sciences, Department of Rehabilitation Sciences, Occupational Therapy, Physiotherapy and Speech-language Pathology/Audiology, Ghent University, Corneel Heymanslaan 10, B3, entrance 46, 9000 Ghent, Belgium; 2 Department of Occupational Therapy, Artevelde University College, Ghent, Belgium; 3 Mental Health Research group, Frailty in Ageing Research Group, Vrije Universiteit Brussel, Brussels, Belgium; 4 Faculty of Psychology and Educational Sciences, Department of Work, Organization and Society, Ghent University, Ghent, Belgium; 5 Department of Physiotherapy (Occupational Therapy), University of Malaga, Málaga, Spain; 6 Psychological Sciences Research Institute, Université catholique de Louvain, Louvain-la-Neuve, Belgium; 7 Department of Experimental Psychology, Ghent University, Ghent, Belgium; 8 Research group on Methodology, Methods, Models and Outcomes of Health and Social Sciences (M3O), Faculty of Health Science and Welfare, University of Vic-Central University of Catalonia (UVIC-UCC), 08500 Vic, Spain; 9 Department of Occupational Therapy. School of Health Sciences, Catholic University of Murcia, Murcia, Spain; 10 Research Group GrEUIT, Escola Universitària d’Infermeria i Teràpia Ocupacional de Terrassa (EUIT), Universitat Autònoma de Barcelona, Terrassa, Spain

**Keywords:** Corona, General health, Meaningful activities, Occupations, Resilience, Well-being

## Abstract

**Background:**

The spread of COVID-19 has affected people’s daily lives, and the lockdown may have led to a disruption of daily activities and a decrease of people’s mental health.

**Aim:**

To identify correlates of adults’ mental health during the COVID-19 lockdown in Belgium and to assess the role of meaningful activities in particular.

**Methods:**

A cross-sectional web survey for assessing mental health (General Health Questionnaire), resilience (Connor-Davidson Resilience Scale), meaning in activities (Engagement in Meaningful Activities Survey), and demographics was conducted during the first Belgian lockdown between April 24 and May 4, 2020. The lockdown consisted of closing schools, non-essential shops, and recreational settings, employees worked from home or were technically unemployed, and it was forbidden to undertake social activities. Every adult who had access to the internet and lived in Belgium could participate in the survey; respondents were recruited online through social media and e-mails. Hierarchical linear regression was used to identify key correlates.

**Results:**

Participants (*N* = 1781) reported low mental health (M = 14.85/36). In total, 42.4% of the variance in mental health could be explained by variables such as gender, having children, living space, marital status, health condition, and resilience (β = −.33). Loss of meaningful activities was strongly related to mental health (β = −.36) and explained 9% incremental variance (R^2^ change = .092, *p* < .001) above control variables.

**Conclusions:**

The extent of performing meaningful activities during the COVID-19 lockdown in Belgium was positively related to adults’ mental health. Insights from this study can be taken into account during future lockdown measures in case of pandemics.

## Background

Coronavirus disease 19 (COVID-19) is a novel infectious disease with an onset in Wuhan City, China, in December 2019 [1]. Since then, COVID-19 has become a pandemic and can cause a severe acute respiratory syndrome that can lead to death [2]. The World Health Organization (WHO) declared the COVID-19 outbreak as a public health emergency of international concern [3]. Governments around the globe implemented highly restrictive, sometimes intrusive measures under rapidly changing epidemiological situations [4].

Epidemic diseases are a permanent threat to global health. Nowadays, many infections such as smallpox and influenza can be prevented by vaccinations, and COVID-19 is not the first epidemic to affect Belgium. In the past, Europe, including Belgium had to deal with (Mexican) influenza, smallpox, and the Black Plague. In recent decades, Belgium was spared life-threatening epidemics such as Severe Acute Respiratory Syndrome (SARS) in 2003, which had very similar symptoms of COVID-19 [5]. This is therefore the first time the majority of the Belgian population has experienced something so manifest and profound.

On March 13, 2020, a lockdown in Belgium was a necessary preventive action to avert the threat of a health crisis [6]. Despite this lockdown in the month of June, 2020, more than 15,000 COVID-19 related deaths were reported for Belgium’s population of about 11 million people, that is, 174 deaths per day per million inhabitants (data retrieved on the first of August, 2020) [7].

Research has shown that epidemics can cause severe and variable psychological effects on people [8, 9]. The growing literature on COVID-19 has already shown that the pandemic and accompanying measures have had a profound effect on–besides health issues–all aspects of society, including mental health [10, 11]. Because mental health under COVID-19 is now among the foremost public health concerns throughout the world, it is also one of the most researched topics [12]. Most of the mental health-related studies during COVID-19 focused on caregivers, such as nurses and physicians, who are on the frontlines of the pandemic responses [13–17] or considered the mental health of COVID-19 patients [18].

COVID-19 and mental health is, to date, a rapidly growing field of research. The results are fragmented and a clear picture is still missing. Several mental health studies on COVID-19 found associations between demographic characteristics and mental health. It seems that younger people (< 30 years, inclusive of students [19, 20]) [13, 21–25]; women [9, 13, 19, 20, 22, 26]; healthcare workers [13, 21]; unemployed people [19]; adults who have children [27, 28]; people living in rural areas [29]; those having cancer or chronic diseases [23]; those living with family member at high risk for COVID-19 [9]; and those living alone [23] have the highest risk to develop mental health problems such as anxiety or depression during the COVID-19 crisis.

So far none of these studies on mental health during COVID-19 took the role or potential influence of one’s daily meaningful activities on adult mental health into account, although the lockdown has had a major impact on daily activities [30] and previous research has shown that daily activities can have a huge impact on one’s mental health [31–36].

Activity engagement has also been associated with lower levels of mental health symptoms due to the meaning people attribute to their daily activities [37–41]. Indeed, activity engagement contributes strongly to humans’ sense of purpose in life and fulfilment of psychological basic needs (e.g. need for autonomy, need to belong, and need for competence) leading to better health [42], well-being [43, 44], and successful aging [45–47]. This suggests that the COVID-19 lockdown and the implied immediate and unprecedented impact on multiple facets of life might influence one’s mental health due to a diminished sense of purpose and/or frustration of these psychological needs. This may be especially true for adults who are undergoing a critical time of interpersonal development, education, family development, and career building. For instance, young adults are at high risk for mental health problems as they reported elevated loneliness and depression even after the first 5 months of exposure to the COVID-19 pandemic [22].

Considering the relevance of meaningful activities for mental health, it can be concluded that there is a gap in the COVID-19 literature about the relationship between mental health and meaningful activities during lockdown. This study therefore examined whether meaning in activities was associated with mental health, regardless of demographic and personal characteristics (e.g., resilience).

The hypothesis (H) of this study was that the inability to engage in meaningful activities during COVID-19 lockdown would be a contributing factor for low mental health. Specifically, we expected a positive association between engaging in meaningful activities and mental health in adults, even after taking control variables into account.

## Materials and methods

### Study design

A cross-sectional online survey was conducted among a sample of adults in Belgium, via the online platform Limesurvey©. The study adhered to the strengthening of the reporting of observational studies in epidemiology (STROBE) statement [48].

### Procedure and participants

The first lockdown measures in Belgium began on March 13, 2020, comprising the following restrictions: (1) primary schools, Universities of Applied Sciences, and Universities had to close and turned digital; (2) all not-essential shops had to close, and only food-related shops were permitted to be open; (3) businesses had to close and employees became technically unemployed or had to work from home; (4) planned activities such as concerts and, sports events were cancelled; (5) recreational settings such as cinemas and, fitness centers, had to close; and (6) it was forbidden to see family and friends outside one’s own house and to undertake social activities. These measures lasted until May 4, 2020, when the number of COVID-19 infections stabilized.

The web-based self-reported questionnaire was distributed online in Belgium (Flanders and Wallonia) between April 24 (6 weeks after elapsing the total lockdown) and May 4, 2020. The data collection stopped after the government introduced a reduction in the measures with a gradual start-up of work, shops, and schools.

Participants were recruited through online communications on social media (Facebook, e-mails, Twitter, and LinkedIn) and through e-mails (sent to the authors’ networks, patient associations, colleges, and universities).

To capture the entire lifespan of adulthood, every adult, aged 18 years or older, who had access to the internet and lived in Belgium, could voluntarily and anonymously participate in the study. Participants under the age of 18 were excluded because they are too young to answer the survey questions properly and are still dependents on their parents.

### Measures

Three standardized and validated measurement instruments were integrated into the online questionnaire and supplemented by additional questions to describe the study sample.

#### Part 1: Sociodemographic and activity related data

The first part of the online questionnaire included operationalizations of the following descriptive variables: age, gender, educational level, marital status, having children, employment status (with working situation), living situation, living region, living area, living space, health condition, and whether the participants were informal caregivers or receiving informal care.

Next, participants were presented a fixed list of 18 daily activities. These activities were relevant across all stages of adulthood and were identified through a literature study including basal, instrumental, and advanced activities of daily living. A consensus was made based on the expertise of the research team, and the activities were linked to the nine domains of the International Classification of Functioning and Disability and Health (ICF) [49].

Participants were asked to indicate whether or not they performed each of these daily activities before the COVID-19 crisis. If so, they had to indicate whether they adapted the activity during COVID-19. Four category options were provided: (1) as usual (in the same manner compared to before the COVID-19 crisis); (2) in another environment (e.g., at home instead of the office); (3) in another way (e.g., online shopping instead of shopping in the street); and (4) another activity evoking the same purpose or meaning (e.g., going for a walk instead of going to the gym). Selecting one or more categories was possible.

#### Part 2: instruments

##### The general health questionnaire (GHQ)

Participants’ mental health was assessed using the 12-item version of the GHQ, which is a valid and reliable instrument for assessing psychological morbidity and distress [50]. This widespread validated instrument is used by the government in (epidemiological) studies in Belgium [51–53]. It had a sensitivity of 83.4% and specificity of 76.3% [54]. Internal consistency of the GHQ in this study sample was calculated with the Cronbach’s Alpha. Every item (e.g., *Lately, have you lose much sleep over worry?*) has four answer options using a Likert scale (0= ‘not at all’; 1= ‘no more than usual’; 2= ‘rather more than usual’; or 3= ‘much more than usual’). The total sum scores can range from 0 to 36, with higher total scores reflecting higher levels of psychological morbidity or distress. A score of 12 or lower in adults indicates psychological well-being [54].

##### The Connor-Davidson resilience scale (CD-RISC)

The CD-RISC measures the personal characteristic of resilience, broadly conceptualized as healthy and adaptive functioning in the aftermath of adversity [55, 56]. The 10-item CD-RISC (e.g., ‘*I am able to adapt when changes occur*’) is scored on a 5-point Likert scale ranging from 0= ‘not true at all’ to 4 = ‘true nearly all of the time’. The total sum score ranges from 0 to 40. A higher score indicates higher resilience [57]. The CD-RISC is a widely recognized and well-validated measurement of resilience in different cultures [58] and populations [59]. In adults, the threshold score is 29 [60]. It showed a good convergent validity, accounted for high explained variance (81.5%), and overall correlation values on divergent validity were close to zero [61]. Internal consistency of the CD-RISC in this study sample was calculated with Cronbach’s Alpha.

##### The engagement in meaningful activities survey (EMAS)

The 12-item EMAS with 4-point Likert scale according to Eakman [62] was used. This measure was developed to assess aspects of activity meaning and emphasizes activity’s congruity with one’s value system and its ability to fulfill basic needs and mastery. The EMAS uses the broad term ‘activities’ to represent typical activities within self-care, leisure, and productivity. Participants were asked to rate each statement (e.g. ‘*The activities I do reflect the kind of person I am’*) on a 4-point Likert scale, ranging from 1 = ‘seldom’ to 4 = ‘always’. A total sum score ranges from 12 to 48. The meaningfulness of a person’s activities can be either low (EMAS < 29), moderate (EMAS 29–41), or high (EMAS > 41). The EMAS was found to have adequate test-retest reliability (r = .71) and significant positive correlations and negative zero-order correlations with measures of meaning and purpose in life, depressive symptomology, life satisfaction, and health-related quality of life [50, 63]. Internal consistency of the EMAS in this study sample was calculated with Cronbach’s Alpha.

### Data analysis

Only full completed questionnaires were used for statistical analyses. All statistics were performed using SPSS 26 (SPSS Inc., Chicago, IL) and a *p* < .05 was considered significant in all analyses. A descriptive analysis (frequencies and means) of the sample (demographics and activities during COVID-19) was conducted.

#### Step 1: bivariate analysis

In preparation for the multivariate analysis and to identify significant correlates of mental health, all potential relevant variables retrieved from the survey were associated with mental health. Bivariate analysis through between-group differences in mental health (GHQ) regarding nominal and ordinal independent variables was evaluated by Mann–Whitney U tests for questions with two answer categories (gender, having children, living area, informal caregiver, receiving care, living space) and Kruskal–Wallis tests for questions with more than two answer categories (marital status, educational level, employment status, living condition, region, working situation, health condition). Mann–Whitney U tests were conducted after the Kruskal–Wallis tests to find which groups significantly differed from each other based on GHQ scores. Spearman’s correlation coefficients were obtained for the continuous independent variables (age, EMAS, and CD-RISC).

#### Step 2: multivariate analysis

To test the hypothesis, multivariate analysis with hierarchical multiple linear regression was used. All categorical variables with more than two answer categories were transformed into ‘dummy variables’. In Model 1, regression coefficients were calculated for the control variables retrieved from bivariate analysis. The factor ‘meaningful activities’ was added in Model 2 to examine whether meaningful activities are statistically significantly related to mental health, while adjusting for key confounding variables (demographic and personal characteristics). Models were screened for multicollinearity according to the calculations of the Variance Inflation Factor (VIF). F-values below 0.10 were automatically removed because can be considered insignificant.

## Results

### Descriptive results

#### Sociodemographic variables

In total, a convenience sample of 1781 participants completed the questionnaire. Descriptive data for the independent variables are displayed in Table 1.

**Table 1:** Overview of sociodemographic characteristics of the study sample (*N* = 1781)

Mean age (SD)	34.79 (16.8)		
		%	N
Gender	Woman	75.3	1341
	Men	24.7	440
Marital status	Married	27.4	489
	Divorced	2.8	50
	Single	37.4	667
	Widow	1.3	23
	Living together	18.8	335
	Other	12.2	217
Educational level	Elementary school	0.6	10
	Middle school	22.8	406
	University of Applied Sciences and University	76.6	1365
Health condition	Healthy	78	1389
	Acutely ill	2.9	52
	Chronically ill	17.9	318
	Mentally ill	1.2	22
Employment status	Student	45	801
	Employed	37.3	664
	Unemployed	17.7	316
Working situation (if employed)	I work from home	26.1	465
	I work as usual	10.9	195
	Technically unemployed	2.6	47
	Other	4.4	79
Having children	Yes	33.7	601
	No	66.2	1180
Living situation	Living alone	9	160
	Living alone with children	2	35
	Living together without children	23.9	425
	Living together with children	18.5	330
	Living with parents, family	41.7	742
	In a student room alone	0.8	15
	In a dorm with another student	2.3	41
Living region	Flanders (Dutch-speaking part)	94.7	1687
	Wallonia (French-speaking part)	3.2	57
	Brussels	2.1	37
Living area	Urban	42.5	757
	Rural	57.5	1024
Living space	House, apartment with garden or balcony	92.1	1640
	Studio, apartment without balcony	7.9	141
Giving informal care	Yes	9.3	166
	No	90.7	1615
Receiving informal care	Yes	6.1	108
	No	93.9	1673

*SD* Standard deviation

#### Activities during the COVID-19 lockdown

An overview of the activities that the participants performed before and during the COVID-19 crisis can be found in Table 2. Thirty-seven percent of the respondents who indicated that they did social activities outside the home before COVID-19 stopped these activities during COVID-19 lockdown. Approximately, two-third of the students who did a student job and nearly 50 % of the adults who volunteered before the COVID-19 crisis had to quit these activities. Activities that continued indoors, alone, or in a ‘bubble’ with closest family, such as leisure time (97.3%), caring for family (99.4%), household (99.3%), doing chores in the house (98%), and self-care (99.8%), were largely retained.

**Table 2:** Percentages of participants who discontinued or maintained the selected activities during COVID-19 lockdown

	‘Don’t do anymore’Participants who discontinued activities	‘Still do’Participants who maintained the activities
Self-care (*n* = 1754)	0.2%	99.8%
Free time activities indoors (*n* = 1697)	2.7%	97.3%
Social activities outdoors (*n* = 1619)	37.4%	62.6%
Household (*n* = 1616)	0.7%	99.3%
Free time activities outdoors (*n* = 1473)	17%	83%
Caring for my health through sports (*n* = 1436)	3.7%	96.3%
Caring for my health through cooking (*n* = 1351)	2.7%	97.3%
Sexuality (*n* = 1255)	13.1%	86.9%
Attending a training (*n* = 945)	4.6%	95.4%
Doing chores in and around the house (*n* = 841)	2%	98%
Going to work (*n* = 792)	6.4%	93.6%
Caring for family (*n* = 515)	0.6%	99.4%
Caring for others outside the family (*n* = 465)	18.5%	81.5%
Volunteering work (*n* = 401)	48.9%	51.1%
Caring for my health through mental activities (*n* = 385)	7%	93%
Student job (*n* = 326)	68.4%	31.6%
Doing chores for other people (*n* = 169)	39.6%	60.4%
Religious activities (*n* = 92)	12%	88%

*n* = number of participants who performed the activity before the COVID-19 crisis

Table 3 shows whether or not participants adapted their activities during the lockdown according to the four described categories. The activities that were performed in-house during the COVID-19 lockdown were hardly modified. In particular, taking care of themselves (61%) and the family (56%), cooking healthy foods (68%), sexual activity (83%), doing household-related activities (79%), doing chores in the house (61%) and performing student jobs (82%) did not happen any differently than prior to the COVID-19 outbreak.

**Table 3:** Percentages of participants who (did not) change(d) their activities during COVID-19

	Not different	In another environment	In another way	Comparable activity
Self-care (*n* = 1750)	61%	15%	22%	16%
Free time activities indoors (*n* = 1651)	44%	29%	22%	29%
Social activities outdoors (*n* = 1015)	11%	40%	70%	36%
Household (*n* = 1603)	79%	7%	13%	7%
Free time activities outdoors (*n* = 1226)	16%	69%	56%	10%
Caring for my health through sports (*n* = 1383)	32%	48%	34%	35%
Caring for my health through cooking (*n* = 1317)	68%	20%	15%	7%
Sexuality (*n* = 1085)	83%	4%	10%	5%
Attending trainings (*n* = 902)	6%	66%	84%	4%
Doing chores in and around the house (*n* = 824)	61%	17%	27%	8%
Going to work (*n* = 741)	23%	65%	31%	2%
Caring for family (*n* = 513)	56%	15%	23%	23%
Caring for others outside the family (*n* = 382)	25%	32%	55%	4%
Volunteering work (*n* = 205)	27%	39%	48%	12%
Caring for my health through mental activities (*n* = 358)	46%	23%	26%	29%
Student job (*n* = 106)	82%	6%	8%	4%
Doing chores for other people (*n* = 102)	37%	24%	40%	18%
Religious activities (*n* = 81)	37%	56%	26%	7%

However, other daily activities such as sports (48%), religion (56%), attending trainings (66%), and going to work (65%) were still performed but largely in a different environment. Adults social outside activities (70%), attending trainings (84%), and caring for others outside the family (55%) were performed in a different manner during COVID-19 lockdown.

Finally, 35% of the participants regularly cared for their health through sports, and 36% of the participants performed social activities prior to the COVID-19 outbreak reported that they replaced these daily activities with other still comparable activities evoking the same purpose or meaning.

#### Mental health, resilience and meaning in activities

Internal consistency of the measurement instruments was calculated for GHQ (Cronbach’s Alpha = .888), EMAS (Cronbach’s Alpha = .907), and CD-RISC (Cronbach’s alpha = .883), and showed a good to excellent internal consistency for every instrument.

The mean GHQ score for the overall sample was 14.85. Based on the threshold score of 12 for adults, the mean score lies above the threshold score which reflects higher levels of psychological morbidity or distress.

Participants scored on average 24.85 on the CD-RISC, which indicates a lower resilience than the cut-off score of 29 for adults.

Based on the EMAS categories, 655 participants experienced low meaning in activities, 1013 participants experienced a moderate sense of meaning in their activities, and 83 experienced high meaning in their activities.

### Bivariate analysis

The independent variables that were statistically significantly associated with mental health (GHQ) in the Mann–Whitney U test were gender, having children, living area, and living space. The results are displayed in Table 4. Woman scored significantly higher in the GHQ than men (*p* < .001), which indicates lower mental health. Participants who had children scored significantly lower in the GHQ than adults without children, which indicates better mental health (*p* < .001). People living in an urban living area had lower mental health than participants who lived in a rural area (*p* = .004). Participants whose living space included a garden or balcony had a lower score in the GHQ which reflects better mental health (*p* < .001).

**Table 4:** Overview bivariate analysis results (1): Mann-Whitney U test: differences between (dichotomous) groups for the General Health Questionnaire (*N* = 1781)

	GHQ Mean (SD)	Mann-Whitney U	Z	P
**Gender**		230,410	−6.909	<.001
Woman	15.44 (6.80)			
Men	13.04 (6.55)			
**Having children**		258,587	−9.364	<.001
Yes	12.89 (6.58)			
No	15.84 (6.72)			
**Living area**		356,404	−2.909	<.05
Rural	14.44 (6.71)			
Urban	15.41 (6.92)			
**Informal caregiver**		123,306	−1.704	.088
Yes	15.65 (6.90)			
No	14.77 (6.80)			
**Receiving care**		81,136	−1.779	.075
Yes	16.14 (7.40)			
No	14.77 (6.77)			
**Living space**		91,852.5	−4.06	<.001
Balcony/garden	14.62 (6.67)			
No balcony/garden	17.50 (7.9)			

Higher scores in GHQ means lower mental health

The significant independent variables associated with mental health and retrieved from the Kruskal-Wallis test were marital status, employment status, living condition, health condition. The results are displayed in Table 5.

**Table 5:** Overview bivariate analysis results (2): Kruskal–Wallis test: differences between (categorical) groups for the General Health Questionnaire followed by Mann–Whitney U (*N* = 1781)

	GHQ Mean (Range; SD)	GHQ Median (inter quartile)	*P*-value Kruskal– Wallis	*P*-value Mann-Whitney U^a^
**Marital status**			<.001	
1.Married	12.54 (0–36; 6.18)	11 (9)		.033 between 1 and 2
2.Divorced	14.74 (3–32; 7.08)	15 (11)		<.001 between 1 and 3
3.Alone	16.56 (1–36; 6.79)	16 (10)		<.001 between 1 and 5
4.Widow	12.91 (1–23; 5.61)	13 (7)		<.001 between 1 and 6
5.Living together	14.17 (0–36; 6.73)	13 (10)		.019 between 3 and 4
6.Other	16.07 (2–35; 6.81)	16 (10)		<.001 between 3 and 5
				.001 between 5 and 6
**Educational level**			.156	
Primary education	12.3 (4–20; 5.74)	10.50 (11)		
Secondary education	15.44 (1–35; 7.17)	14.50 (11)		
Higher education	14.69 (0–36; 6.71)	14 (10)		
**Employment status**			<.001	
1.Student	16.67 (1–36; 6.80)	16 (9)		<.001 between 1 and 2
2.Employed	13.16 (0–34; 6.25)	12 (9)		<.001 between 1 and 3
3.Not employed	13.78 (1–36; 6.90)	12 (9)		
**Living condition**			<.001	
1.Alone	15.19 (2–35; 7.30)	14 (11)		.002 between 1 and 3
2.Alone with children	14.29 (1–29; 6.97)	15 (10)		.001 between 1 and 4
3.Living together without children	13.09 (0–36; 6.23)	12 (9)		.025 between 1 and 5
4.Living together with children	12.83 (0–36; 6.47)	11.5 (9)		.001 between 1 and 7
5.Living in with parent(s),family	16.36 (1–36; 6.50)	16 (10)		.004 between 2 and 7
6.Room alone	19.27 (5–36; 8.37)	21 (12)		<.001 between 3 and 5
7.Room together	19.34 (3–35; 8.01)	22 (13)		.003 between 3 and 6
8.Other	14.88 (3–34; 7.96)	13 (11)		<.001 between 3 and 7
				<.001 between 4 and 5
				.002 between 4 and 6
				<.001 between 4 and 7
				.005 between 5 and 7
				.025 between 7 and 8
**Region**			.545	
Flanders	14.88 (0–36; 6.80)	14 (11)		
Wallonia	14.81 (3–34; 7.53)	14 (11)		
Brussels	13.68 (2–32; 6.95)	12 (10)		
**Working situation**			.504	
Working from home	13.36 (0–33; 6.44)	13 (10)		
Work as usual	12.92 (4–30; 5.46)	11 (8)		
Technically unemployed	14.74 (4–34; 7.54)	13 (10)		
Other	14.28 (0–36; 7.58)	13 (8)		
**Health condition**			<.001	
1.Healthy	14.63 (0–36; 6.66)	14 (10)		.011 between 1 and 2
2.Acute ill	17.02 (2–31; 6.99)	16 (11)		.001 between 1 and 4
3.Chronically ill	15.06 (3–36; 7.09)	14 (10)		.042 between 2 and 3
4.Mentally ill	20.23 (2–36; 9.17)	20.50 (11)		.003 between 3 and 4

To increase the interpretability, means as well as medians were displayed in this table. Higher scores in GHQ indicate lower mental health. ^a^The numbers in the last column correspond with the groups described in column 1

Married respondents scored significantly lower in the GHQ than being divorced (*p* = .033), being alone (*p* < .001), and living together (*p* < .001), which indicates better mental health.

Being alone scored significantly lower in the GHQ than being a widow (*p* = .019) or living together (*p* < .001), meaning better mental health.

Students scored significantly higher in the GHQ than employed (*p* < .001) and unemployed adults (*p* < .001), indicating worse mental health.

Living alone scored significantly higher in the GHQ than living together with (*p* = .001) and without children (*p* = .002), meaning lower mental health.

Participants who lived with parent(s) or family scored higher in the GHQ than those living alone (*p* = .025), living together (with children (*p* < .001) or without children (*p* < .001)), meaning lower mental health.

Participants who lived in a room together with other people (e.g., student accommodation) scored higher in the GHQ than participants who lived alone (*p* = .001), alone with children (*p* = .004), or those living together (with children (*p* < .001) or without children (*p* < .001)) or living in with parent(s) or family (*p* = .005), meaning lower mental health.

Participants who lived in a room alone scored higher in the GHQ than participants who lived together, with children (*p* = .002), meaning lower mental health.

Healthy participants scored lower in the GHQ than acutely ill participants (*p* = .011) or mentally ill participants (*p* < .001), which indicates better mental health. Chronically ill persons scored lower in the GHQ than mentally ill participants (*p* = .003) and acutely ill participants (*p =* .042), meaning better mental health.

As shown in Table 6, Spearman’s Rho test showed a significant moderate relationship between EMAS-GHQ (Rs = −.547, *p* < .001) and CD-RISC-GHQ (Rs = −.524, *p* < .001). Age was also significantly related to mental health (Rs = −.254, *p* < .001).

**Table 6:** Overview bivariate analysis (3): Spearman correlation test: associations between mental health and continuous independent variables in study sample (*N* = 1781)

	Spearman’s Rho	P
EMAS	−.547	<.001
CD-RISC	−.524	<.001
Age	−.254	<.001

VIF factors showed no problems in terms of multicollinearity, except for ‘living condition’ and ‘marital status’, which reflect both whether or not respondents live alone or share their life with someone. The decision based on a VIF factor of 1 was to exclude living condition. A closer look at the variables ‘age’, ‘employment status’ and ‘having children’ showed that they intrinsically indicate the same thing, namely the stage of life. The correlations between age and employment status (*r* = .792), age and having children (*r* = .753) were strong. Accordingly, the decision was made to exclude ‘age’ in the following analysis.

### Multivariate analysis

In the next step, mental health was regressed using all at once predictors that were statistically significantly related to mental health in the bivariate analysis.

Results from the hierarchical regression analysis are provided in Table 7. The eight control variables (gender, having children, living area, living space, employment status, marital status, health condition, and resilience (CD-RISC)) were entered simultaneously into Model 1 and together accounted significantly for 33.2% of the variance in mental health (*p* < .001). In Model 2, the variable meaning in activity (EMAS) was added to the regression equation, which explained 9.2% incremental variance, augmenting the total explained variance in mental health to 42.4% (*p* < .001). Six of the eight control variables were significantly associated with mental health, including gender (β = −.038*; p* = .043), having children (β = −.055; *p* = .015), living space (β = −.040*; p* = .035), marital status (β = −.076; *p* = .001), health condition (β = −.054; *p* = .006), and resilience (β = −.332; *p* < .001).

**Table 7:** Hierarchical regression analysis of demographic characteristics, personal factors and meaning in activities associated with mental health

		Mental health				
	Model 1			Model 2		
	B	SE B	β	B	SE B	β
Control variables						
Gender	−.793	.317	−.050*	−.596	.294	−.038*
Having children	−.968	.353	−.067*	−.798	.328	−.055*
Living area	.391	.279	.028	.254	.259	.018
Living space	−1.164	.512	−.046*	− 1.002	.476	−.040*
Employment status	−.297	.314	−.021	−.083	.292	−.006
Marital status	−1.536	.331	−.112**	−1.037	.309	−.076*
Health condition	−.975	.344	−.059*	−.884	.319	−.054*
Resilience (CD-RISC)	−.530	.021	−.501**	−.351	.022	−.332**
Main effect						
EMAS				−.363*	.022	−.358**
R^2^			.335**			.427**
Adjusted R^2^			.332**			.424**
R^2^ change			.335**			.092**

**significant result (*p* < .001), *significant result (*p* < .05)

## Discussion

To gain insight into the mental health of Belgian adults during the COVID-19 lockdown, this study investigated some potential predictors of mental health and focused on the role of meaning in activities in particular. Based on our results, we could confirm our hypothesis that meaningful activities do contribute to general mental health in adults during COVID-19 lockdown.

The COVID-19 pandemic caused significant changes to peoples’ daily lives as their movements were restricted in an effort to slow down the spread of the virus. Faced with new realities of working from home, online meetings, temporary unemployment, home-schooling (of children), and lack of social contact with friends and family, the mental health of people was affected [64].

Based on the discussion of Allsop and colleagues [65], the number of people suffering from mental health problems during a major event is often greater than the number of people suffering from physical problems. The results of our study showed low mental health among Belgian adults, which is not unexpected as current COVID-19 research showed similar results elsewhere. Using the same instrument, GHQ, researchers from the United Kingdom Household Longitudinal Study (UKHLS) [66] did a comparison between mental health pre-COVID-19 and during COVID-19, they found that adults had worse mental health during COVID-19 than pre-COVID-19. A follow-up study found that, in the first COVID-19 wave 29.2% of their respondents scored above the threshold on the GHQ, which indicates cases of psychiatric disorder [67]. Furthermore, from April 2020 until June 2020, all sociodemographic groups showed significant increases in mental health problems in the UK [68].

The loss of mental health can, according to our study results, be partly explained by the loss of meaning in activities. Hierarchical regression showed that meaning in activities (EMAS) accounted incrementally for 9.2% of the variance in mental health (GHQ), beyond the effects of demographic and personal variables. The subjective experience of meaning associated with engagement in activity may be a key mechanism through which daily activities influence personal well-being and, according to the study results, mental health [69–71]. As stated, meaning in activities is a key outcome of human health and well-being and our study demonstrated that this also holds true during a pandemic, when anxiety and stress prevail [42, 44].

As the forced adaptation to the lockdown measures likely caused an unintended disruption or even (temporary) loss of daily activities (e.g., work, hobbies) for most people (e.g., children, adults, and elderly), one might expect that day-to-day living, behavior, and well-being might be blurred [4, 66]. People were forced to adapt their daily activities, and activities that could not easily be adapted — such as volunteering work, student jobs, and most affected of all, social activities outdoors — had to be discontinued [72]. At the start of the lockdown, people experienced the measures as a temporary disruption of the situation and adapted by trying to find new ways to experience meaning in the performed activities by changing the form of the activity, searching for other activities, or changing their time investment in these activities [73]. As the lockdown persisted, people struggled to find meaning and started to attribute the negative effects of the measures to features beyond their control (e.g., people started blaming the government that the measures were too hard). We thus assume, based on an occupational therapy model, that the temporary disruption turned into experiences of deprivation in which the people were restricted from participation in necessary meaningful activities due to circumstances outside their control [74].

It can be presumed that the longer the COVID-19 pandemic lasted, the more difficult it was to remain satisfied with curtailed activities, and the more people lost meaning in these activities. Due to the imposed measures, the form in which people were forced to perform their activities was reduced to basic functions (e.g. the function of shopping is to ensure oneself of the necessary ingredients to stay alive) [73]. People became task-oriented, yet were less likely to engage in creative thinking which is primarily driven primarily by enjoyment and a sense of meaning in activities [75, 76]. Consequently, the form of the activities did not leave enough room for aspects of meaning, such as shopping together for fun. This could thus be an explanation of why most of the participants experienced moderate meaning in activities. Comparing this result with a normative adult group that scored high in meaning in activities under normal circumstances (M = 45,3), we can state that the COVID-19 lockdown caused a serious loss in meaning in activities for Belgian adults [51]. Resilience was the second strongest contributor to mental health after meaning in activity. These results are in line with other recent studies. In the face of COVID-19, people need to cope with these ongoing stressors and minimize psychological distress [77]. Being resilient can protect against these events and make it possible to thrive from adversity [78–80]. In a COVID-19 study across healthcare and non-healthcare professionals, higher resilience scores were associated with lower COVID-19 related worries, as well as a reduced rate of anxiety and depression [14].

The demographic factors gender, having children, marital status, living space, and current health condition contributed statistically significantly to lower mental health in our study sample. Women had lower mental health than men in this study, which is in line with other COVID-19 studies of mental health in women [13, 19, 20, 22]. Females’ sense of identity in activities is more affected by the social connections formed, rather than by other aspects in the activity setting [81]. Women seem to attach more meaning to social activities, which could hardly or no longer be carried out during COVID-19 lockdown, while men attach more meaning to physical activities that could still be performed during lockdown. The care and household tasks, in which women are usually more active, interfered much more with other activities than usual during lockdown. In Belgium, parenting adults could request corona leave to manage the care of (home-schooled) children. Seventy-five percent of the parents who claimed the leave appeared to be women [82]. In general, women were found to be more susceptible to mental health related symptoms than men because they show greater emotional response [27]. It can thus be concluded that women were found to be slightly more vulnerable in terms of mental health than men.

It seems that being married during the COVID-19 lockdown helped respondents to have better mental health. There is a considerable amount of evidence showing that married people have better mental health than never-married and divorced people [83, 84], which can be explained by the importance of the satisfaction and support associated with such a dedicated relationship [85]. Our study results suggest that living alone may be related to lower mental health during lockdown compared to adults who are widows or living together. Previous research has also confirmed that living with a partner is a protective factor for general psychiatric disorders during the COVID-19 crisis [67].

Next, our study results demonstrated that chronically ill adults need to have specific attention. They are less affected in terms of mental health during the COVID-19 lockdown compared to mentally and acutely ill adults. Moreover, it is remarkable that chronically ill persons did not statistically significantly differ from healthy people. They may have been better able to adapt to the changing situation in COVID-19 lockdown because they already experienced a loss of activity and prolonged isolation due to their illness, thus preparing them to be better able to cope with this crisis [86, 87].

To our knowledge, living space has not yet been described in the COVID-19 literature as a contributor to mental health. During the COVID-19 lockdown, the strict measures prohibited outdoor activities: people only could leave the house to provide for the necessities of life or to work if telecommuting was not possible. People who lived with access to a garden or balcony, could go outside and do physical or psychological activities there. It is already proven that (physical) activities in an outdoor space can protect against mental health problems, and restricted access is likely to have negative implications for mental health [88–96]. People without the opportunity to go outside their house (e.g. no garden or balcony) might have experienced deprivation, which could cause the lower mental health we found in this study. Similar results were found in an epidemiological study where the type of housing children inhabited altered mental health correlates [97]. High-rise multiple dwelling units are inimical to the mental health of mothers with young children because of the social isolation and restricted play opportunities for children [97]. A study in low-income housing areas in London showed that having less access to private gardens was associated with a higher prevalence of depression [98].

Although we found a statistically significant bivariate association between living area (rural versus urban) and mental health in our study sample of adults, it was not a significant contributor for mental health in the multivariate analysis. This suggests that its explanatory power is limited compared to the other included predictors. Living in a rural area was associated with better mental health during the COVID-19 lockdown than living in an urban area, perhaps because it provides opportunities to take long walks outdoors and, be in nature, which positively promotes mental health [88–96]. However, this is contradicted in the study of Summer-Gabr [99] in the United States, where living in urban areas appeared to show better mental health than people living in rural areas due to better access to health care and mental health services. We speculate that the meaning and potential impact of a variable such as living area on mental health might be weakened in our study as Belgium is a very small country in which the difference between rural and urban areas is less manifest. This implies that other factors, such as resilience and one’s engagement in meaningful activities, might have a greater influence on mental health.

Finally, participants without children had lower mental health than adults with children. This is contrary to the common findings in the current literature. A national COVID-19 study in the US explained the low mental health in parents by the fact that parents reported worsening behavioral health for their children [28] and unstable financial circumstances, school closure, suspended educational services for children may underlie low mental health in parents [100]. Our contrary results could be due to the fact that most of the participants who did not have children in our study, were students who do not belong to the working adults group.

An unexpected result is that work status was not significantly associated with mental health in the multivariate analysis, despite a bivariate association. COVID-19 studies have already shown that unemployed adults and students have a greater chance of being exposed to mental health problems [19, 67]. Similarly, in our study, students reported lower mental health compared to employed and non-employed adults, and the latter two groups did not differ substantially in terms of mental health. These results might reflect that students were a vulnerable subgroup during COVID-19 lockdown. Alternatively, employment, as measured in our study, might be too generic a variable, which neglects the type of job one performs or its characteristics. It might be that mental health is more influenced by the extent of remote working and its interference with daily functioning at home.

Remarkably, educational level was not significantly related to mental health in our study. This contradicts previous studies that have shown people with higher degrees (above bachelor’s level) experienced an increase in mental health problems at the time of the pandemic [26, 68]. Our result might be due to range restriction, as the majority (76.6%) of our participants held a higher educational degree (University of Applied Sciences or University).

Nevertheless, our study demonstrated that maintaining or reconstructing meaningful activities during the COVID-19 pandemic seemed to be an important pathway to build a meaningful life in the context of profound disruption [101]. Increasing awareness of the importance of meaningful activities in daily life might lead to more widespread recognition of the potentially detrimental effects of disruption and deprivation of such activities when these occur [102].

The impact that isolation due to COVID-19 lockdown has on people’s ability to achieve a sense of occupational balance, defined as a balance of engagement in occupations that leads to well-being [103], is alarming. Occupational therapists thus have a critical role to play in helping people respond to this type of crisis [104], and leaders in the profession have articulated its relevance in supporting mental health and wellbeing in populations worldwide [101, 105, 106]. Occupational therapists have already learned a lot from their clients about how everyday life might be rebuilt within constraining parameters that are out of the client’s control. Our unique occupational focus can be central in identifying new and creative solutions in response to the unprecedented and challenging COVID-19 crisis, as we have the right knowledge and the creativity to respond to the massive impact of the COVID-19 pandemic on our clients and the general population [104]. Providing meaningful activities during social lockdown is an important point of attention that, in addition to health and economic aspects, should be taken into account when deciding on measures concerning COVID-19.

To our knowledge, this is one of the first national sample studies to consider a comprehensive set of factors that might contribute to adults’ mental health, and in particular, identified meaningful activities as a plausible key predictor of mental health during times of global crisis.

## Conclusion

The findings suggest that sociodemographic factors, resilience, and meaning in activities were associated with reduced mental health. Each of the seven identified factors has to be taken into account to emerge from lockdown in a sustainable way and from a social perspective, to maintain mental health. This study stressed the importance of activities during COVID-19 and raises a new idea that could be interesting for health policymakers during and beyond COVID-19: besides economical and health aspects, meaning in activities must also be taken into account throughout lockdown decision-making processes and measures.

### Methodological considerations / limitations

The presented results need to be interpreted with caution owing to some study limitations. First, because this study was initiated during the COVID-19 pandemic, we could not rely on data from the Belgian population regarding resilience, mental health, and meaningful activities from the period before the pandemic. As a consequence, comparisons were not possible and we were unable to capture changes or trajectories in mental health or any of the identified predictors.

Second, the GHQ-12 failed to be a clinical assessment in this study. Results should be interpreted with caution and do not mean that people with low mental health have a mental illness [66].

Third, as our findings are based on a cross-sectional research design, no causal statements can be inferred. Although the present study has shown that there is a strong positive relationship between meaningful activities and mental health in Belgian adults, further longitudinal studies are needed to investigate potential causal effects.

Fourth, as the participants in our sample are not completely representative of the whole Belgium population, future researchers might investigate the generalizability of the study findings to other target groups and countries as well.

Fifth, exclusively self-reported measures were used in this study. These measures can cause common method variance, which can influence results [107]. The authors of this study did, however, take precautions to reduce this possible effect by indicating that there were no right or wrong answers, by guaranteeing anonymity, and by using existing valid scales with sufficient internal consistency. Future research might incorporate ratings from multiple sources (e.g., peer ratings) or assess more objective indicators and measures, in addition to subjective measures. Finally, another drawback of a self-reported online survey is that participants who have questions cannot address them directly to the researcher. To give participants the opportunity to raise questions we did include, however, an open field question at the end of the survey (last question), for this purpose, and the contact details of the first author of this study were given at the start of the survey and participants were encouraged to e-mail with questions and comments regarding the survey as well.

## Data Availability

The datasets used and/or analyzed during the current study are available from the corresponding author on reasonable request.

## Abbreviations

COVID-19: Coronavirus Disease
WHO: World Health Organization
SARS: Severe Acute Respiratory Syndrome
H: Hypothesis
STROBE: Strengthening the Reporting of Observational Studies in Epidemiology
ICF: International Classification of Functioning, Disability and Health
GHQ: General Health Questionnaire
CD-RISC: Connor-Davidson Resilience Scale
EMAS: Engagement in Meaningful Activities Survey
VIF: Variance Inflation Factor
UKHLS: United Kingdom Household Longitudinal Study
